# The role of green tea intake in thromboprophylaxis of venous thromboembolism in patients with cancer

**DOI:** 10.3389/fnut.2024.1296774

**Published:** 2024-05-02

**Authors:** Qihuan Yao, Hongwei Qiao, Yi Cheng, He Du, Yanbin Zhang, Yong Luo, Hongwei Wang, Song Liu, Mei Xu, Wei Xiong

**Affiliations:** ^1^Department of Traditional Chinese Medicine, Kongjiang Hospital, Shanghai, China; ^2^Department of Medical Oncology, Kongjiang Hospital, Shanghai, China; ^3^Department of Pulmonary and Critical Care Medicine, Xinhua Hospital, Shanghai Jiaotong University School of Medicine, Shanghai, China; ^4^Department of Medical Oncology, Shanghai Pulmonary Hospital, Tongji, University School of Medicine, Shanghai, China; ^5^Department of TCM Dermatology, Kongjiang Hospital, Shanghai, China; ^6^Department of Pulmonary and Critical Care Medicine, Chongming Hospital, Shanghai University of Medicine and Health Science, Shanghai, China; ^7^Department of General Practice, North Bund Community Health Service Center, Shanghai, China; ^8^Department of Cardiovascular Medicine, Graduate School of Medicine, Kyoto University, Kyoto, Japan

**Keywords:** green tea, venous thromboembolism, prophylaxis, cancer, antiplatelet

## Abstract

**Background:**

Green tea intake has been reported to improve the clinical outcomes of patients with cardiovascular diseases or cancer. It may have a certain role in the development of venous thromboembolism (VTE) among cancer patients. The current study aimed to address this issue, which has been understudied.

**Methods:**

We carried out a retrospective study to explore the role of green tea intake in cancer patients. Patients with and without green tea intake were enrolled in a 1:1 ratio by using propensity scoring matching. The primary and secondary outcomes were VTE development and mortality 1 year after cancer diagnosis, respectively.

**Results:**

The cancer patients with green tea intake (*n* = 425) had less VTE development (10 [2.4%] vs. 23 [5.4%], *p* = 0.021), VTE-related death (7 [1.6%] vs. 18 [4.2%], *p* = 0.026), and fatal pulmonary embolism (PE) (3 [0.7%] vs. 12 [2.8%], *p* = 0.019), compared with those without green tea intake (*n* = 425). No intake of green tea was correlated with an increase in VTE development (multivariate hazard ratio (HR) 1.758 [1.476–2.040], *p* < 0.001) and VTE-related mortality (HR 1.618 [1.242–1.994], *p* = 0.001), compared with green tea intake. Patients with green tea intake less than 525 mL per day had increased VTE development (area under the curve (AUC) 0.888 [0.829–0.947], *p* < 0.001; HR1.737 [1.286–2.188], *p* = 0.001) and VTE-related mortality (AUC 0.887 [0.819–0.954], *p* < 0.001; HR 1.561 [1.232–1.890], *p* = 0.016) than those with green tea intake more than 525 mL per day. Green tea intake caused a decrease in platelet (*p* < 0.001) instead of D-dimer (*p* = 0.297). The all-cause mortality rates were similar between green tea (39 [9.2%]) and non-green tea (48 [11.3%]) intake groups (*p* = 0.308), whereas the VTE-related mortality rate in the green tea intake group (7 [1.6%]) was lower than that of the non-green tea intake group (18 [4.2%]) (*p* = 0.026). The incidences of adverse events were similar between the green tea and non-green tea intake groups.

**Conclusion:**

In conclusion, the current study suggests that green tea intake reduces VTE development and VTE-related mortality in cancer patients, most likely through antiplatelet mechanisms. Drinking green tea provides the efficacy of thromboprophylaxis for cancer patients.

## Introduction

Venous thromboembolism (VTE) that comprises both pulmonary embolism (PE) and deep vein thrombosis (DVT) affects nearly 10 million people every year globally. The annual incidence of acute VTE is 1–2 cases per 1,000 population. Approximately 20% of patients with PE die within 1 year of diagnosis, mostly due to comorbidities such as cancer rather than recurrent PE (1–4). VTE in cancer patients (that is usually termed cancer-associated VTE) is a common and life-threatening condition. The presence of cancer increases the risk for VTE by approximately ninefold, especially in cancer patients who undergo chemotherapy. VTE is the leading cause of death in cancer patients, second only to cancer itself. The occurrence of VTE increases the likelihood of death in cancer patients by approximately two- to sixfold. Appropriate VTE prophylaxis can bring about substantial benefits for cancer patients who are at VTE risk (5, 6).

A recent research revealed an antithrombotic signature of heat shock protein 47 (HSP47) as the most substantially reduced protein in platelets of hibernating brown bears. The down-regulation or ablation of HSP47 attenuates immune cell activation and neutrophil extracellular trap formation, thereby contributing to thromboprophylaxis in bears, paralyzed spinal cord injury patients, and mice (7). Epigallocatechin-3-gallate (EGCG), which is the most important component of green tea catechins (GTC) in green tea, has been identified as a potent inhibitor of HSP47 and is proven to be beneficial for the treatment of PE in a murine model (8). In a two-sample Mendelian randomization study carried out to explore whether there is a causal association between green tea intake and arterial embolism and thrombosis in the MRC-IEU consortium (64,949 participants with green tea intake) and the FinnGen project (278 cases of arterial thrombosis and 92,349 control participants), genetically determined green tea intake was causally associated with a lower risk of arterial embolism and thrombosis (9). Green tea intake is also considered to have prophylactic and therapeutic efficacy in cancer (10, 11).

Taken together, since green tea containing EGCG having antiplatelet effect by inhibiting HSP47, thereby yielding the effect of thromboprophylaxis, has been verified in mouse models of PE and patients with arterial thrombosis, it was hypothesized that green tea intake may have a prophylactic role in the development of VTE among cancer patients. Due to the high incidence of VTE occurrence, recurrence, and mortality, cancer patients would be benefited, provided that it was determined that drinking green tea had certain prophylactic efficacy on VTE development through a study. To our best knowledge, there is no such literature to date. Therefore, the current study was performed to address this issue.

## Methods

### Study design

We conducted a retrospective study to investigate the role of green tea in prophylaxis in the development of VTE among cancer patients, whose initial diagnosis was made at least 1 year prior to the current study. Depending on whether patients drank brewed green tea or not, all eligible patients were classified into the green tea group and non-green tea group. Patients in the non-green tea group were defined as those who never or only occasionally drank green tea, whereas patients in the green tea group were defined as those who had a habit of drinking green tea everyday (12). In the green tea group, patients were subdivided into subgroups according to the average volume of green tea they drank per day. Based on the previous literature (9), all patients in the green tea group were classified into subgroup 1 (0–250 mL per day), subgroup 2 (250–500 mL per day), and subgroup 3 (500 mL or more per day) in the present study, according to the self-reported average volume of green tea that they drank per day. In China, tea brewing is typically conducted by infusing 2–3 grams of loose tea leaves in a cup containing 100–150 mL hot water with a temperature of 85–100°C. Only the volume of the first and second tea infusions was counted if there was more subsequent water refill since the GTC and EGCG for the third tea infusion in hot water significantly declined (12–15). The minimum unit of measurement for green tea intake was 50 mL.

In line with the recommendation in guidelines (16), VTE risk assessment was conducted routinely every 3 months after the diagnosis of cancer. During each VTE risk assessment, for patients with clinical VTE symptoms or signs, or a D-dimer level of 1,000 ng/mL or more, computed tomography pulmonary angiography (CTPA), compression ultrasonography (CUS) of lower extremities, and/or planar ventilation/perfusion (V/Q) scan were conducted to objectively diagnose or exclude VTE. Patients with a sudden clinical VTE symptom or signs at the hospital or home were also instructed to be evaluated for VTE risk and undergo VTE imaging investigation if necessary. In general, pharmacological thromboprophylaxis was implemented for patients in surgery, systemic anticancer treatment, and hospitalization, unless there was a contraindication to anticoagulation. All patients underwent anticancer treatment according to the requirements of their condition. The specific decisions for prophylactic anticoagulation and anticancer treatment were at the discretion of the patients’ attending physicians.

The primary outcome was VTE development 1 year after the cancer diagnosis/index date. The secondary outcome was mortality 1 year after the cancer diagnosis/index date including all-cause and VTE-related mortality. VTE-related mortality was defined as patients for whom the underlying cause of death was PE or VTE (17). The occurrence of VTE and mortality was recorded on a monthly basis. The overall prevalence of VTE development and all-cause and VTE-related mortality 1 year after cancer diagnosis were compared between green tea and non-green tea groups. Time-dependent cumulative VTE incidence and all-cause and VTE-related mortality rates 1 year after cancer diagnosis were compared between green tea and non-green tea groups. The correlation between green tea intake and outcomes was analyzed. The predictive power of volume of green tea intake per day for outcomes in the green tea group was analyzed. Comparisons of decrease in platelet as well as D-dimer from cancer diagnosis to 1 year after cancer diagnosis or death within 1 year after cancer diagnosis were conducted between green tea and non-green tea groups. Subgroup analysis with respect to outcomes was conducted among subgroups 1, 2, and 3 in the green tea group. We also documented possible adverse events due to green tea intake in the green tea group, compared with the same adverse events in the non-green tea group. According to previous literature (14, 18), we mainly focused on hepatotoxicity, which was defined as an increase of alanine aminotransferase (ALT), aspartate aminotransferase (AST), gamma glutamyltransferase (GGT), alkaline phosphatase (AP), or bilirubin in the blood concentration, and gastrointestinal irritation, which was defined as vomiting and/or diarrhea.

The present study was undertaken by medical researchers of Shanghai Kongjiang Hospital, Shanghai Xinhua Hospital, Shanghai Pulmonary Hospital, Chongming Hospital of Shanghai University of Medicine and Health Science, and Shanghai North Bund Community Health Service Center. The data required for analysis were obtained mainly through the electronic medical record system or telephone communication. All authors contributed more or less to the writing of the manuscript. All authors vouch for the completeness, fidelity, and accuracy of the data. All authors have read and approved the submitted version of the manuscript for publication. Patients or the public were not involved in the design, or conduct, or reporting, or dissemination plans of our research. This study protocol was approved by the Institutional Review Board of each participating hospital.

### Study population

We collected eligible patients according to the inclusion and exclusion criteria. Inclusion criteria comprised the following: (1) patients were 18 years old or older; (2) patients were objectively diagnosed with primary active cancer (19) for the first time at least 1 year prior to the current study; (3) patients underwent VTE risk assessment periodically, as well as VTE imaging investigation and thromboprophylaxis if necessary. Exclusion criteria were as follows: (1) patients already had an objectively confirmed chronic thromboembolic disease (CTED) or acute VTE prior to the diagnosis of cancer; (2) patients underwent a concurrent use of single or dual antiplatelet therapies besides prophylactic anticoagulation; (3) patients drank other kinds of tea apart from green tea concurrently; (4) patients had more than one primary active cancer.

### Statistical analysis

Propensity score matching was adopted in the present study to offset the bias of potential confounding factors highly associated to cancer-associated VTE. Based on the predictors in risk assessment scores of cancer-associated VTE (5, 6, 20), we matched age, body mass index (BMI), previous VTE, and familial and/or acquired hypercoagulability due to reasons other than cancer, mainly including thrombophilia, active autoimmune diseases, nephrotic syndrome, oral contraception, and pregnancy; medical comorbidities including infection, renal disease, pulmonary disease, congestive heart failure, or arterial thromboembolism, primary site of cancer, performance status, and metastasis; VTE-related anticancer therapies including major surgery, chemotherapy, immunotherapy, protein kinase inhibitors (PKI), hormonal or antiangiogenic therapies, central venous catheter (CVC), hospitalization, or prolonged immobilization; and use of red cell growth factors, platelet, hemoglobin, leukocyte, D-dimer, Khorana score, and thromboprophylaxis between green tea and non-green tea groups. Nearest-neighbor (greedy) matching without replacement was adopted in the propensity score matching analysis (21). We adopted a 1:1 ratio by using a caliper width of 0.2 of pooled standard deviation of the logit of the propensity score (22) for the number of patients between non-green tea and green tea groups. The matching algorithm selected one patient in the green tea group first and then selected one patient in the non-green tea group who had a linear propensity score being closest to that of the selected one in the non-green tea group.

Comparison of measurement data was performed by using *t*-test or analysis of variance. Comparison of rates was performed by using the chi-square test. Time-dependent cumulative incidence of VTE development, all-cause, and VTE-related mortality rates between green tea and non-green tea groups was explored by using Kaplan–Meier curve analysis. The correlation between outcomes and green tea intake as well as other risk factors of cancer-associated VTE (5, 16, 20) including age, BMI, previous VTE, hypercoagulability, medical comorbidities, primary site of cancer, performance status, metastasis, VTE-related anticancer therapies, CVC, hospitalization or prolonged immobilization, platelet, use of red cell growth factors, hemoglobin, leukocyte, D-dimer, Khorana score, and thromboprophylaxis were analyzed by using the multivariate Cox proportional hazard model. The predictive efficiency of the volume of green tea intake per day for VTE development was analyzed by using receiver operator characteristic (ROC) curve analysis. We used SPSS 26 and R software 3.6.1 (R Project for Statistical Computing) for statistical analyses. A *p*-value less than 0.05 denotes statistical significance.

## Results

### Characteristics of patients

A total of 1,521 patients from the participating hospitals between 2012 and 2022 who met the inclusion criteria were enrolled. Then, 1,312 patients remained in the study after the exclusion of 209 patients according to the exclusion criteria. Among these 209 patients, 25 patients already had an objectively confirmed CTED or acute VTE prior to the diagnosis of cancer, 74 patients underwent a concurrent use of antiplatelet therapies besides prophylactic anticoagulation, 97 patients concurrently drank other kinds of tea rather than green tea, and 13 patients had more than one primary active cancer. After the following propensity score matching, a total of 850 cancer patients entered into the final analysis, in which the number of patients in green tea and non-green tea groups were 425 and 425, respectively. The number of patients in subgroups 1, 2, and 3 were 139, 151, and 135, respectively. The median age of all patients was 69.8 years. Among them, 422 patients were women, whereas 428 patients were men. The cancer types included prostate, breast, lung, lymphoma, gynecologic, bladder, brain, stomach, and pancreatic. The VTE-related anticancer therapies mainly included major surgery, chemotherapy, immunotherapy, protein kinase inhibitors (PKIs), hormonal therapies, and antiangiogenic therapies. The agents used for thromboprophylaxis were low molecular weight heparin (LMWH), direct oral anticoagulant (DOAC), and warfarin. The median length of green tea drinking history was 25.2 years in the green tea group, whereas it was 27.6, 25.8, and 22.2 years in the subgroups 1, 2, and 3, respectively. The median volume of green tea intake per day was 336.9 mL/day in the green tea group, whereas it was 152.6, 408.7, and 711.8 mL/day in the subgroups 1, 2, and 3, respectively. The demographics and characteristics of enrolled patients before and after propensity score matching are demonstrated in Tables 1, 2, respectively.

**Table 1:** Demographics and characteristics of patients before propensity score matching.

Variables	Green tea (*n* = 609)	Non-green tea (*n* = 703)	*p*-value
Age-years	67.3 ± 19.6	70.7 ± 21.4	0.136
Sex (female/male)-no. (%)	303(49.8)/306(50.2)	361(51.4)/342(48.6)	0.564
BMI-kg/m^2^	23.1 ± 6.9	22.8 ± 5.2	0.353
Previous VTE (Y/N)-no. (%)	51(8.4)/558(91.6)	118(16.8)/585(83.2)	<0.001
Hypercoagulability (Y/N)—no. (%)	85(14.0)/524(86.0)	185(26.3)/518(73.7)	<0.001
Medical comorbidities (Y/N)—no. (%)	294(48.3)/315(51.7)	422(60.0)/281(40.0)	<0.001
Primary site of cancer-no. (%)			
Prostate Breast Lung Lymphoma Gynecologic Bladder Brain Stomach Pancreas Others	60(9.9) 62(10.2) 89(14.6) 77(12.6) 85(14.0) 26(4.3) 27(4.4) 76(12.5) 51(8.4) 56(9.2)	99(14.1) 101(14.4) 88(12.5) 72(10.2) 80(11.4) 48(6.8) 17(2.4) 91(12.9) 55(7.8) 52(7.4)	0.019 0.022 0.268 0.171 0.160 0.045 0.043 0.801 0.715 0.237
Performance status-points	2.0 ± 1.3	2.2 ± 1.6	0.336
Metastasis (Y/N)—no. (%)	253(41.5)/356(58.5)	401(57.0)/302(53.0)	<0.001
VTE-related anticancer therapies-no. (%)			
Major surgery Chemotherapy Immunotherapy Protein kinase inhibitor Hormonal therapies Antiangiogenic therapies	212(34.8) 406(66.7) 112(18.4) 253(41.5) 81(13.3) 97(15.9)	175(24.9) 512(72.8) 137(19.5) 316(45.0) 89(12.7) 121(17.2)	<0.001 0.015 0.613 0.214 0.730 0.533
CVC (Y/N)—no. (%)	244(40.1)/365(59.9)	301(43.2)/402(57.2)	0.313
Hospitalization or prolonged immobilization (Y/N)—no. (%)	422(69.3)/187(30.7)	512(72.8)/191(27.2)	0.158
Use of red cell growth factors (Y/N)—no. (%)	188(30.9)/421(69.1)	222(31.6)/481(68.4)	0.782
Hemoglobin-g/L	128.2 ± 43.4	122.3 ± 38.7	0.561
Platelet- × 10^9^/L	196.7 ± 108.3	225.4 ± 119.1	0.003
Leukocyte- × 10^9^/L	7.4 ± 3.9	7.7 ± 4.1	0.737
D-dimer-mg/L	1.3 ± 1.6	1.7 ± 1.8	0.139
Khorana score-points	1.2 ± 1.5	2.1 ± 1.9	0.036
Thromboprophylaxis-no. (%)			
Overall LMWH DOAC Warfarin	500(82.1) 421(69.1) 54(8.9) 25(4.1)	610(86.8) 514(73.1) 73(10.4) 23(3.3)	0.019 0.112 0.354 0.423

no., number; BMI, body mass index; kg/m^2^, kilogram/meter^2^; Y/N, yes/no; VTE, venous thromboembolism; CVC, central venous catheterization; L, liter; g/L, gram/liter; mg/L, milligram/liter; LMWH, low molecular weight heparin; DOAC, direct oral anticoagulant. Medical comorbidities denote infection, renal disease, pulmonary disease, congestive heart failure, or arterial thromboembolism at the diagnosis of cancer. Since the same patient may receive multiple anticancer therapies, the sum of the various VTE-related anticancer therapies may be greater than 100%. The variables at the diagnosis of cancer were adopted for age, BMI, PS, metastasis, hemoglobin, platelet, leukocyte, D-dimer, and Khorana score.

**Table 2:** Demographics and characteristics of patients after propensity score matching.

Variables	Green tea (*n* = 425)	Non-green tea (*n* = 425)	*p*-value
Age-years	71.2 ± 22.5	68.4 ± 18.1	0.512
Sex (female/male)-no. (%)	204 (48.0)/221(52.0)	218 (51.3)/207(48.7)	0.337
BMI-kg/m^2^	22.7 ± 5.1	21.4 ± 4.6	0.459
Previous VTE (Y/N)-no. (%)	45(10.6)/380(89.4)	42(9.9)/383(90.1)	0.734
Hypercoagulability (Y/N)—no. (%)	80(18.8)/345(81.2)	85(20.0)/340(80.0)	0.665
Medical comorbidities (Y/N)—no. (%)	235(55.3)/190(44.7)	228(53.6)/197(46.4)	0.630
Primary site of cancer-no. (%)			
Prostate Breast Lung Lymphoma Gynecologic Bladder Brain Stomach Pancreas Others	49(11.5) 52(12.2) 55(12.9) 44(10.4) 51(12.0) 22(5.2) 18(4.2) 50(11.8) 37(8.7) 47(11.1)	47(11.1) 54(12.7) 52(12.2) 43(10.1) 56(13.2) 20(4.7) 15(3.5) 48(11.3) 40(9.4) 50(11.8)	0.828 0.836 0.756 0.910 0.605 0.752 0.594 0.830 0.720 0.746
Performance status-points	2.1 ± 1.5	1.9 ± 1.3	0.549
Metastasis (Y/N)—no. (%)	222(52.2)/203(47.8)	208(48.9)/217(51.1)	0.337
VTE-related anticancer therapies-no. (%)			
Major surgery Chemotherapy Immunotherapy Protein kinase inhibitor Hormonal therapies Antiangiogenic therapies	140(32.9) 301(70.8) 88(20.7) 171(40.2) 60(14.1) 77(18.1)	144(33.9) 295(69.4) 85(20.0) 167(39.3) 65(15.3) 72(16.9)	0.771 0.653 0.798 0.779 0.628 0.652
CVC (Y/N)—no. (%)	198(46.6)/227(53.4)	193(45.4)/232(54.6)	0.731
Hospitalization or prolonged immobilization (Y/N)—no. (%)	308(72.5)/117(27.5)	303(71.3)/122(28.7)	0.703
Use of red cell growth factors (Y/N)—no. (%)	146(34.4)/279(65.6)	140(32.9)/285(67.1)	0.663
Hemoglobin-g/L	125.3 ± 37.1	121.8 ± 33.6	0.906
Platelet- × 10^9^/L	218.9 ± 96.1	206.4 ± 107.8	0.212
Leukocyte- × 10^9^/L	7.7 ± 4.4	7.2 ± 4.8	0.972
D-dimer-mg/L	1.6 ± 1.4	1.4 ± 1.2	0.162
Khorana score-points	1.8 ± 1.7	1.9 ± 1.5	0.708
Thromboprophylaxis-no. (%)			
Overall LMWH DOAC Warfarin	372(87.5) 310(72.9) 44(10.4) 18(4.2)	366(86.1) 304(71.5) 47(11.1) 15(3.5)	0.543 0.646 0.739 0.594

no., number; BMI, body mass index; kg/m*^2^*, kilogram/meter^2^; Y/N, yes/no; VTE, venous thromboembolism; CVC, central venous catheterization; L, liter; g/L, gram/liter; mg/L, milligram/liter; LMWH, low molecular weight heparin; DOAC, direct oral anticoagulant. Medical comorbidities denote infection, renal disease, pulmonary disease, congestive heart failure, or arterial thromboembolism at the diagnosis of cancer. Since the same patient may receive multiple anticancer therapies, the sum of the various VTE-related anticancer therapies may be greater than 100%. The variables at the diagnosis of cancer were adopted for age, BMI, PS, metastasis, hemoglobin, platelet, leukocyte, D-dimer, and Khorana score.

### Primary and secondary outcomes

The overall number of patients with VTE development, all-cause mortality, and VTE-related mortality 1 year after cancer diagnosis was 33 (3.9%), 87 (10.2%), and 25 (2.9%) in all 850 patients, respectively. The VTE development rate in the green tea group (10 [2.4%]) was lower than that in the non-green tea group (23 [5.4%]) (*p* = 0.021). The proportion of PE (4 [0.9%] vs. 6 [1.4%], *p* = 0.525), DVT (3 [0.7%] vs. 8 [1.9%], *p* = 0.129), and PE with DVT (3 [0.7%] vs. 9 [2.1%], *p* = 0.081) was similar between green tea and non-green tea groups, respectively. The PE fatality rate in the green tea group (3 [0.7%]) was lower than that in the non-green tea group (12 [2.8%]) (*p* = 0.019). The symptomatic VTE in the green tea and non-green tea groups was 6 (1.4%) and 13 (3.1%), respectively (*p* = 0.104). The incidental VTE in the green tea and non-green tea groups was 4 (0.9%) and 10 (2.4%), respectively (*p* = 0.106). The between-group comparison of secondary outcomes demonstrated that the all-cause mortality rates were similar between green tea (39 [9.2%]) and non-green tea (48 [11.3%]) groups (*p* = 0.308), whereas the VTE-related mortality in the green tea group (7 [1.6%]) was lower than that in the non-green tea group (18 [4.2%]) (*p* = 0.026).

**Figure 1:**
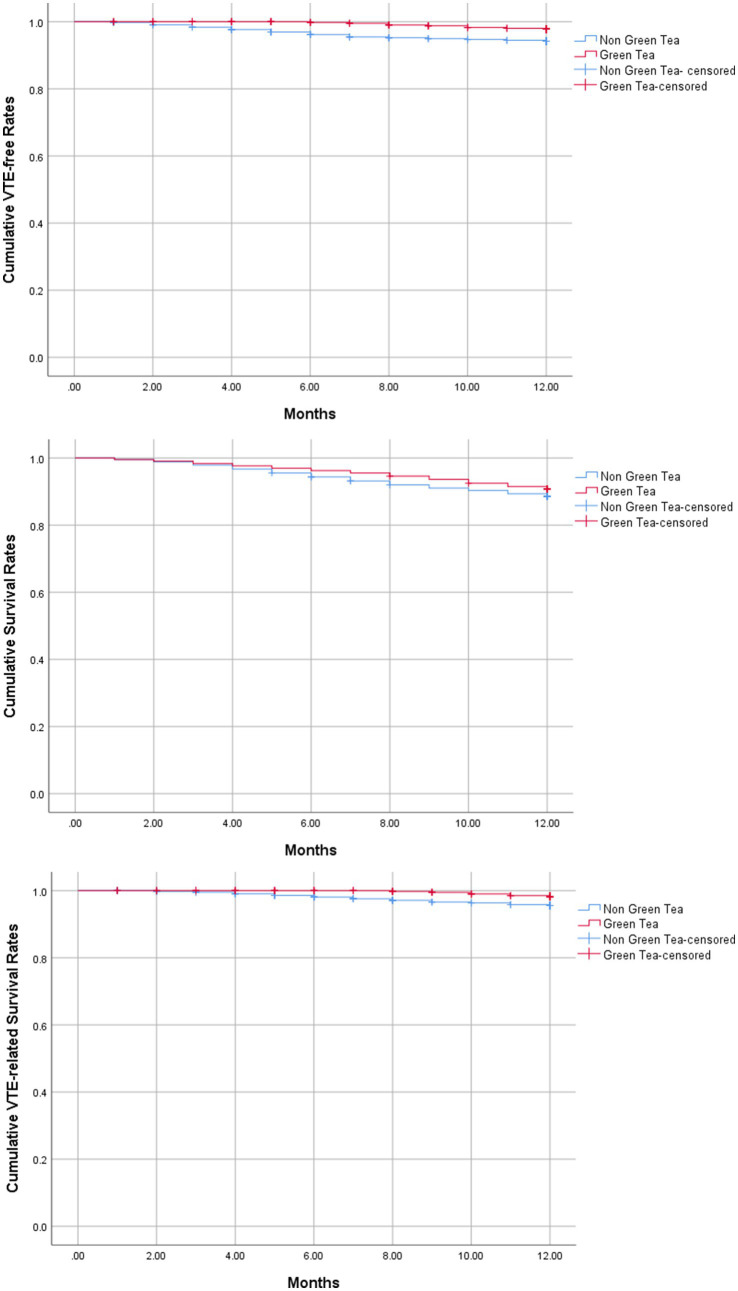
Time-dependent cumulative VTE development and mortality. VTE, venous thromboembolism.

By using a Kaplan–Meier analytical method, the time-dependent cumulative VTE-free incidence (*p* = 0.007) and VTE-related survival incidence (*p* = 0.023) during 1 year after cancer diagnosis in the non-green tea group declined more dramatically than those in the green tea group, whereas the time-to-event overall survival rates were similar between the two groups (*p* = 0.283). The time-dependent cumulative VTE development and mortality are demonstrated in Figure 1.

### Correlation between green tea intake and outcomes

After a sequential univariate and multivariate Cox regression analysis, the green tea intake (HR 1.758 [1.476–2.040], *p* < 0.001), previous VTE (HR 1.737 [1.305–2.169], *p* = 0.001), hypercoagulability (HR 1.824 [1.336–2.312], *p* < 0.001), medical comorbidities (HR 1.616 [1.317–1.915], *p* = 0.003), performance status (HR 1.603 [1.233–1.973], *p* = 0.002), metastasis (HR 1.785 [1.416–2.154], *p* < 0.001), VTE-related anticancer therapies (HR 1.935 [1.410–2.460], *p* < 0.001), CVC (HR 1.702 [1.327–2.077], *p* = 0.001), platelet (HR 1.506 [1.207–1.805], *p* = 0.002), D-dimer (HR 1.863 [1.453–2.273], *p* < 0.001), Khorana score (HR 1.645 [1.298–2.039], *p* = 0.001), and thromboprophylaxis (HR 1.712 [1.227–2.197], *p* < 0.001) were finally correlated with VTE development. Another multivariate Cox analysis showed that green tea intake was also correlated with VTE-related mortality (HR 1.618 [1.242–1.994], *p* = 0.001). The correlation between VTE predictors and VTE development is shown in Table 3.

**Table 3:** Correlation between VTE development and green tea intake as well as other risk factors.

Predictors	Univariate-HR (95%CI)	*P*-value	Multivariate-HR (95%CI)	*P*-value
No green tea intake (green tea intake as 1.0)	1.701(1.325–2.077)	0.001	1.758(1.476–2.040)	<0.001
Age (age < 65 years old as 1.0)	1.205(1.007–1.401)	0.538		
BMI (BMI < 35 kg/m^2^ as 1.0)	1.218(1.016–1.420)	0.462		
Previous VTE (no previous VTE history as 1.0)	1.669(1.347–1.991)	0.001	1.737(1.305–2.169)	0.001
Hypercoagulability (no hypercoagulability as 1.0)	1.931(1.427–2.435)	<0.001	1.824(1.336–2.312)	<0.001
Medical comorbidities (no medical comorbidities as 1.0)	1.552(1.234–1.870)	0.005	1.616(1.317–1.915)	0.003
Primary site of cancer (cancer of high VTE risk as 1.0)	1.443(1.135–1.752)	0.017	1.229(1.038–1.421)	0.134
PS (PS < 2 points as 1.0)	1.664(1.247–2.081)	0.001	1.603(1.233–1.973)	0.002
Metastasis (no metastasis as 1.0)	1.772(1.391–2.153)	0.001	1.785(1.416–2.154)	<0.001
VTE-related anticancer therapies (no VTE-related anticancer therapies as 1.0)	1.836(1.351–2.321)	<0.001	1.935(1.410–2.460)	<0.001
CVC (no CVC as 1.0)	1.651(1.226–2.076)	0.004	1.702(1.327–2.077)	0.001
Hospitalization or prolonged immobilization (no hospitalization or prolonged immobilization as 1.0)	1.508(1.212–1.806)	0.016	1.332(1.114–1.550)	0.075
Platelet (platelet<350 × 10^9^/L as 1.0)	1.695(1.248–2.142)	0.001	1.506(1.207–1.805)	0.002
Hemoglobin (hemoglobin>100 g/L as 1.0)	1.223(1.008–1.438)	0.538		
Use of red cell growth factors (no use of red cell growth factors as 1.0)	1.447(1.159–1.735)	0.012	1.261(1.042–1.481)	0.646
Leukocyte (leukocyte<11 × 10^9^/L as 1.0)	1.315(1.142–1.661)	0.025	1.139(1.025–1.367)	0.773
D-dimer (D-dimer<1.44 mg/L as 1.0)	2.058(1.675–2.441)	0.001	1.863(1.453–2.273)	<0.001
Khorana score (Khorana score < 2 as 1.0)	1.531(1.258–1.804)	0.010	1.645(1.298–2.039)	0.001
No thromboprophylaxis (thromboprophylaxis as 1.0)	1.696(1.234–2.158)	0.001	1.712(1.227–2.197)	<0.001

VTE, venous thromboembolism; HR, hazard ratio; CI, confidence interval; BMI, body mass index; kg/m*^2^*, kilogram/meter^2^; PS, performance status; CVC, central venous catheterization; L, liter; g/L, gram/liter; mg/L, milligram/liter. Medical comorbidities denote infection, renal disease, pulmonary disease, congestive heart failure, or arterial thromboembolism at the diagnosis of cancer. VTE-related anticancer therapies denote major surgery, chemotherapy, immunotherapy, protein kinase inhibitor, hormonal therapies, and antiangiogenic therapies.

### Predictive efficiency of volume of green tea intake per day for outcomes

**Figure 2:**
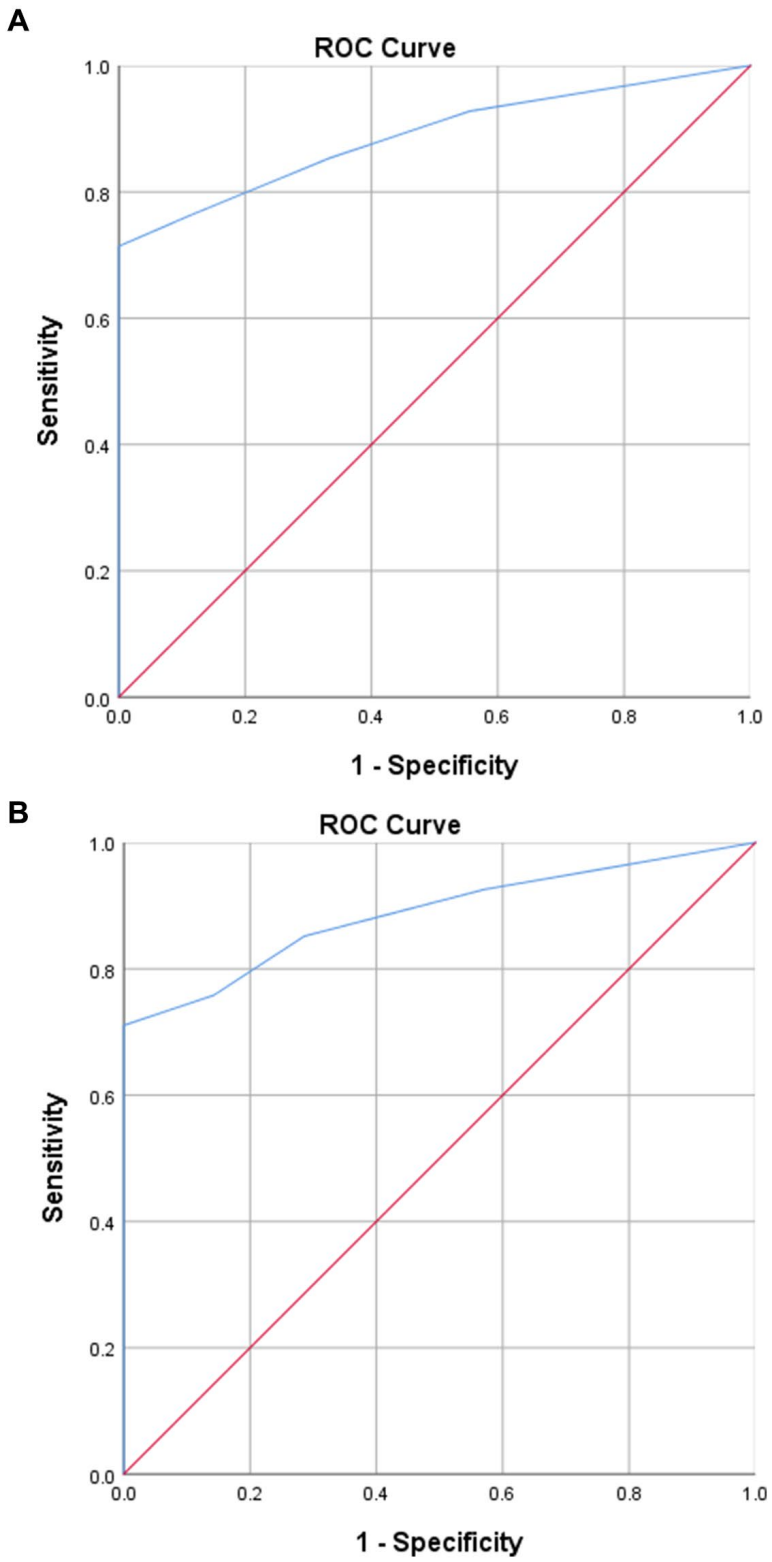
Predictive efficiency of green tea intake per day for outcomes. **(A)** Predictive efficiency of the volume of green tea intake per day for VTE development in the green tea group. **(B)** Predictive efficiency of the volume of green tea intake per day for VTE-related mortality in the green tea group.

By using an ROC curve analysis, the predictive efficiency of the volume of green tea intake per day for VTE development in the green tea group was explored. The sensitivity and specificity of the volume of green tea intake per day for VTE development were 71.4 and 88.9%, respectively. The area under the curve (AUC) was 0.888 (0.829–0.947) (*p* < 0.001). The cutoff value was 525 mL of green tea per day. It indicates that, for cancer patients who drink green tea, patients with green tea intake of more than 525 mL per day are less likely to develop VTE than those with green tea intake of less than 525 mL per day. The sensitivity and specificity of the volume of green tea intake per day for VTE-related mortality were 71.1 and 85.7%, respectively. The AUC was 0.887 (0.819–0.954) (*p* < 0.001). The cutoff value was 525 mL of green tea per day. It indicates that, for cancer patients who drink green tea, patients with green tea intake of more than 525 mL per day had less VTE-related death than those with green tea intake of less than 525 mL per day. In the green tea group, taking green tea intake of more than 525 mL per day as 1.0, the hazard ratio for a green tea intake of less than 525 mL per day to develop VTE and VTE-related death were (1.737 [1.286–2.188], *p* = 0.001) and (1.561 [1.232–1.890], *p* = 0.016), respectively. The predictive efficiency of the volume of green tea intake per day for outcomes is demonstrated in Figure 2.

### Comparison of decrease of platelets and D-dimer between green tea and non-green tea groups

The results of the comparison of decrease of platelets as well as D-dimer from cancer diagnosis to 1 year after cancer diagnosis or death within 1 year after cancer diagnosis between green tea and non-green tea groups demonstrated that the decrease of platelets was more in the green tea group (55.6 ± 26.3) than in the non-green tea group (29.3 ± 13.1) (*p* < 0.001), whereas that of D-dimer was similar between the green tea (−0.8 ± 0.7) and non-green tea (−1.0 ± 0.8) groups (*p* = 0.297).

### Subgroup analysis

The overall number of patients with VTE development in the subgroups 1 (*n* = 139), 2 (*n* = 151), and 3 (*n* = 135) were 7(5.0%), 2(1.3%), and 1(0.7%), respectively. The pairwise subgroup analysis among subgroups 1, 2, and 3 revealed that the VTE incidence of subgroup 1 was similar to that of subgroup 2 (*p* = 0.069), whereas it was higher than that of subgroup 3 (*p* = 0.035). The VTE incidence of subgroup 2 was similar with that of the subgroups 3 (*p* = 0.629). The all-cause mortality in the subgroups 1, 2, and 3 was 16 (11.5%), 13 (8.6%), and 10 (7.4%), respectively. The pairwise subgroup comparison demonstrated that the all-cause mortality was similar among subgroups 1, 2, and 3 (1vs2, *p* = 0.411; 1vs3, *p* = 0.247; 2vs3, *p* = 0.709). The VTE-related mortality in the subgroups 1, 2, and 3 was 5 (3.6%), 1 (0.7%), and 1 (0.7%), respectively. The pairwise subgroup comparison demonstrated that the VTE-related mortality was similar among subgroups 1, 2, and 3 (1vs2, *p* = 0.079; 1vs3, *p* = 0.106; 2vs3, *p* = 0.937).

The synergistic effect of the prophylactic anticoagulation was also analyzed, although the proportion of patients who did not receive prophylactic anticoagulation was very small. The overall VTE incidence were 6/372 (1.6%), 16/366 (4.4%), 4/53 (7.5%), and 7/59 (11.9%), in the patients with prophylactic anticoagulation and green tea intake, those with prophylactic anticoagulation without green tea intake, those with green tea intake without prophylactic anticoagulation, and those without green tea intake or prophylactic anticoagulation (1.6% vs. 4.4%, *p* = 0.028; 1.6% vs. 7.5%, *p* = 0.008; 1.6% vs. 11.9%, *p* < 0.001; 4.4% vs. 7.5%, *p* = 0.311; 4.4% vs. 11.9%, *p* = 0.018; 7.5% vs. 11.9%, *p* = 0.443), respectively.

### Adverse events

The incidence of hepatotoxicity was 142 (33.4%) and 151 (35.5%) in the green tea and non-green tea groups, respectively (*p* = 0.516). The incidence of gastrointestinal irritation was 257 (60.5%) and 233 (54.8%) in the green tea and non-green tea groups, respectively (*p* = 0.096). There was no statistical disparity either, with regard to adverse events among subgroups 1, 2, and 3 in the green tea group.

## Discussion

In the current study, cancer patients with green tea intake had less VTE development, VTE-related death, and fatal PE, compared with those without green tea intake, despite green tea intake not affecting all-cause mortality. No green tea intake was correlated with an increase in VTE development, compared with green tea intake. The volume of green tea intake per day had predictive power for VTE development. An intake volume of less than 525 mL per day was associated with an increase in VTE development or VTE-related death, compared with an intake volume of more than 525 mL per day, in the green tea group. The green tea intake caused a decrease in platelet instead of D-dimer. Subgroup 3 had less VTE development than subgroup 1. Adverse events were similar in patients with and without green tea intake. To our best knowledge, the present study is unprecedented, for no comparable studies are available.

The patient population in the present study consisted of a large variety of cancer types, mainly including prostate and breast cancers, being consistent with the cancer distribution in the general population for years (23), as well as lung, lymphoma, gynecologic, bladder, brain, stomach, and pancreatic cancers, which well-represented cancers with high or very high thromboembolic risk (5). In other words, the current patient population not only has a general representation of cancer patients but also includes cancer patients who are at high risk of VTE, thereby avoiding a selection bias. The overall VTE morbidity in the present patient population was 3.9%, being similar to that of a large population-based cohort study including 499,092 patients with a first-time cancer diagnosis and 1,497,276 individuals without cancer, in which the cumulative incidence of VTE 12 months after the cancer diagnosis/index date was 2.3% (24). Taken together, the current patient population makes the results of the study have an excellent generalizability.

Despite the role of green tea intake in the development of VTE among cancer patients being understudied, its role in cardiovascular diseases or cancer has been thoroughly studied. In a population-based, prospective cohort study among 40,530 Japanese adults without history of stroke, coronary heart disease, or cancer at baseline, green tea consumption was associated with reduced all-cause and cardiovascular disease-related mortality, but not with reduced mortality due to cancer (12). In a systematic review and meta-analysis including 22 prospective studies among 856,206 individuals, increased tea consumption was associated with reduced risk of coronary heart disease, cardiac death, stroke, cerebral infarction, intracerebral hemorrhage, and total mortality (25).

With respect to thrombotic diseases, it was revealed that GTC and EGCG prevented death caused by pulmonary thrombosis for mice *in vivo* in a dose-dependent manner. The modes of antithrombotic action may be due to antiplatelet activities instead of anticoagulation ones (26). In addition to GTC, EGCG, or EGC, CsCPI1, which is a cysteine proteinase inhibitor (CPI) from green tea, also promotes antithrombotic activity by inhibiting platelet aggregation (27). Furthermore, epigallocatechin (EGC) in green tea was demonstrated to have both significant anticoagulation and antiplatelet activities *in vivo* in another research (28). In the current study, green tea intake manifested antiplatelet efficacy instead of anticoagulation, being consistent with the rationale mentioned in the *Introduction*. Since the anticancer treatments that can cause thrombocytopenia were balanced between the two groups, plus all patients received no antiplatelet therapy, the significantly higher decrease in platelet among patients in the green tea group than that in the non-green tea group suggests that green tea may have certain antiplatelet effects. Although not the first choice, aspirin is also considered for thromboprophylaxis in cancer patients, justifying that the antiplatelet effect of green tea may be beneficial to the prophylaxis of cancer-associated VTE (6, 16, 29). By contrast, no significant difference of D-dimer change was observed between the two groups. From the clinical perspective of the efficacy of green tea intake on thrombotic diseases, only a study provided causal evidence between genetically predicted green tea intake and the prophylaxis of arterial embolism and thrombosis (9). The present study is the first one that provides clinical evidence for the prophylactic efficacy of green tea intake in the development of VTE among cancer patients.

In addition to antiplatelet efficacy, whether the anticancer efficacy of green tea may facilitate the decrease of VTE incidence among cancer patients in the present study deserves further discussion. Anticancer efficacy of green tea still remains relatively controversial, despite an already academic viewpoint that green tea has anticancer effects (10, 11). Green tea did not reduce the mortality due to cancer in the Ohsaki Study (12). In a population-based, prospective cohort study in Japan, no association between green tea consumption and the risk of gastric cancer was observed (13). In a meta-analysis, overall findings from epidemiological studies yielded inconsistent results, thereby providing limited evidence for the beneficial effect of green tea consumption on the overall risk of cancer or specific cancer sites (30). Likewise, we observed no improvement due to green tea intake in overall outcomes of cancer patients in the present study.

Another issue is the interaction of green tea intake with other medicine. It was reported that green tea is rich in vitamin K and thus may antagonize the efficacy of warfarin (31). In the present study, the VTE incidence among the patients undergoing warfarin therapy for thromboprophylaxis was similar between the green tea and non-green tea groups. In addition, the number of patients receiving warfarin was considerably small (<5%) in the present study, and there were no reports of interaction of green tea intake with LMWH or DOAC in previous literature. Similarly, no evidence was found that green tea intake affected the efficacy of LMWH and DOAC in this study.

With regard to adverse events, no statistical difference was observed between the green tea and non-green tea groups. Although we focused on hepatotoxicity and gastrointestinal irritation which may frequently occur in cancer patients due to chemotherapy, immunotherapy or targeted therapy, since those anticancer treatment have been balanced between the two groups by using propensity score matching, green tea intake seemed to add no further impact on the adverse events. The present study suggested that a minimum of 525 mL per day may be an optimal volume of green tea intake for the thromboprophylaxis of VTE in cancer patients. According to the United States Department of Agriculture (USDA) Flavonoid Database, brewed green tea usually contains an average of 126.6 mg total catechins and 77.8 mg EGCG per 100 mL (14). As such, the optimal EGCG per day for VTE thromboprophylaxis in the current study is estimated to be 408.5 mg, which is below the safe intake limit of green tea, that is, 704 mg EGCG per day in a beverage form (14, 32).

The clinical implications of the current results lie in some aspects. First, clinicians could encourage cancer patients to drink green tea for its potential VTE prophylactic efficacy as well as reduction in VTE-related death. Second, for cancer patients with green tea intake, 525 mL green tea intake per day or more may be the optimal volume for VTE thromboprophylaxis. Third, drinking green tea contributes little to improvement of cancer conditions. Fourth, the prophylactic function for VTE from green tea intake is mainly through its antiplatelet efficacy rather than the anticoagulant one. Fifth, the combination of prophylactic anticoagulation and green tea drinking can provide the optimal prophylactic effect against VTE, being superior to the isolated prophylactic anticoagulation, the isolated green tea drinking, and neither of them, whereas only drinking green tea without prophylactic anticoagulation may not have much benefit. Last, drinking green tea seldom has adverse events or interaction with other medicine.

### Strengths and limitations

The current study has certain strengths. First, the introduction of propensity score matching into the current study contributed to a favorable comparability between the green tea and non-green tea groups, thereby minimizing the influence of bias and confounding factors on the results to the greatest extent. Second, we excluded patients who concurrently drank other kinds of tea than green tea to ensure that disparity with respect to thromboprophylaxis between the two groups was due to isolated green tea drinking. Last, we also observed the disparity of changes in platelets and coagulation indicators between the two groups, which clarified the major mechanism of thromboprophylaxis of green tea intake to a certain extent. The result confirmed the hypothesis in the *Introduction* section.

Nevertheless, it is still necessary to acknowledge some limitations in the current study. First, since it was a retrospective study, a prospective one is warranted in the future, despite that propensity score matching may eliminate the bias of confounding factors to some degree. Second, the daily volume and adherence of green tea intake were orally self-reported by the patients or their family in the green tea group, whereas no daily green tea drinking record was documented. Nevertheless, for patients with a fixed habit of green tea drinking, drinking tea is their routine daily compulsory course. Third, it was impracticable to understand the actual concentration of GTC or EGCG in the green tea intake per day, despite the volume of green tea intake being collected. However, the cups and volume of green tea drinking were always adopted in the relevant studies (12, 13). In addition, the brands of green tea varied from patient to patient. Nevertheless, no report of difference in GTC or EGCG among different green tea brands has been found to our best knowledge. Fourth, the propensity score matching in the present study mainly targeted cancer-associated VTE instead of mortality, the risk factors of mortality may not be matched between green tea and non-green tea groups, as well as among subgroups in the green tea group. Fifth, we only reported hepatotoxicity and gastrointestinal irritation, which are most frequent and critical adverse events resulted from green tea intake in the present study. Some adverse events which are infrequent and mild may be overlooked. Sixth, we only calculated the volume of the first and second tea infusions for certain tea leaves since they have the most GTC and EGCG. The results might have been different, provided that we also calculated the volume of refill water after the first and second infusions. Seventh, since most patients in the green tea group had a long green tea drinking habit for dozens of years, the results may be inapplicable to those who just started green tea drinking. Relevant studies may be warranted in the future. Last, since the follow-up period in the present study was 1 year after cancer diagnosis, the current results may only be applicable to such an interval in cancer patients.

## Conclusion

In conclusion, based on the hypothesis that green tea intake may have certain prophylactic effect on VTE development in cancer patients by antiplatelet mechanisms, the current study confirmed that green tea intake did reduce the VTE development and VTE-related mortality in cancer patients through such a mechanism, on the basis of prophylactic anticoagulation, after the analyses of a cohort of cancer patients for the first time. The clinical application of this finding in the future may contribute to the reduction of VTE development and VTE-related death, as well as further improvement of prognosis in cancer patients. Since the current study was a retrospective one, a randomized controlled trial is warranted in the future.

## Data availability statement

The original contributions presented in the study are included in the article/supplementary material, further inquiries can be directed to the corresponding author.

## Ethics statement

The studies involving humans were approved by the Institutional Review Boards of Shanghai Kongjiang Hospital (approval number: LL-2020-KY-14), Shanghai Xinhua Hospital (approval number: XHEC-QT-2021-027). The studies were conducted in accordance with the local legislation and institutional requirements. The ethics committee/institutional review board waived the requirement of written informed consent for participation from the participants or the participants’ legal guardians/next of kin because written informed consent is waived for retrospective study according to relevant ethic regulations in China.

## Author contributions

QY: Conceptualization, Data curation, Formal analysis, Funding acquisition, Investigation, Methodology, Project administration, Resources, Software, Validation, Visualization, Writing – original draft, Writing – review & editing. HQ: Data curation, Formal analysis, Investigation, Methodology, Resources, Software, Validation, Visualization, Writing – review & editing. YC: Data curation, Formal analysis, Funding acquisition, Investigation, Methodology, Resources, Software, Validation, Visualization, Writing – original draft. HD: Data curation, Formal analysis, Investigation, Methodology, Software, Validation, Visualization, Writing – review & editing. YZ: Data curation, Formal analysis, Investigation, Methodology, Software, Validation, Visualization, Writing – review & editing. YL: Data curation, Formal analysis, Investigation, Methodology, Software, Validation, Visualization, Writing – review & editing. HW: Data curation, Formal analysis, Investigation, Methodology, Software, Validation, Visualization, Writing – review & editing. SL: Methodology, Resources, Software, Validation, Visualization, Writing – review & editing, Data curation, Formal analysis, Investigation. MX: Data curation, Formal analysis, Investigation, Methodology, Project administration, Software, Validation, Visualization, Writing – review & editing. WX: Conceptualization, Data curation, Formal analysis, Funding acquisition, Investigation, Methodology, Project administration, Resources, Software, Supervision, Validation, Visualization, Writing – original draft, Writing – review & editing.
